# Role of Transposable Elements in Gene Regulation in the Human Genome

**DOI:** 10.3390/life11020118

**Published:** 2021-02-04

**Authors:** Arsala Ali, Kyudong Han, Ping Liang

**Affiliations:** 1 Department of Biological Sciences, Brock University, St. Catharines, ON L2S 3A1, Canada; aa18uw@brocku.ca; 2 Department of Microbiology, Dankook University, Cheonan 31116, Korea; kyudong.han@gmail.com; 3 Center for Bio-Medical Engineering Core Facility, Dankook University, Cheonan 31116, Korea; 4 Centre of Biotechnologies, Brock University, St. Catharines, ON L2S 3A1, Canada

**Keywords:** transposable elements, mobile elements, gene regulation, evolution, human

## Abstract

Transposable elements (TEs), also known as mobile elements (MEs), are interspersed repeats that constitute a major fraction of the genomes of higher organisms. As one of their important functional impacts on gene function and genome evolution, TEs participate in regulating the expression of genes nearby and even far away at transcriptional and post-transcriptional levels. There are two known principal ways by which TEs regulate the expression of genes. First, TEs provide cis-regulatory sequences in the genome with their intrinsic regulatory properties for their own expression, making them potential factors for regulating the expression of the host genes. TE-derived cis-regulatory sites are found in promoter and enhancer elements, providing binding sites for a wide range of trans-acting factors. Second, TEs encode for regulatory RNAs with their sequences showed to be present in a substantial fraction of miRNAs and long non-coding RNAs (lncRNAs), indicating the TE origin of these RNAs. Furthermore, TEs sequences were found to be critical for regulatory functions of these RNAs, including binding to the target mRNA. TEs thus provide crucial regulatory roles by being part of cis-regulatory and regulatory RNA sequences. Moreover, both TE-derived cis-regulatory sequences and TE-derived regulatory RNAs have been implicated in providing evolutionary novelty to gene regulation. These TE-derived regulatory mechanisms also tend to function in a tissue-specific fashion. In this review, we aim to comprehensively cover the studies regarding these two aspects of TE-mediated gene regulation, mainly focusing on the mechanisms, contribution of different types of TEs, differential roles among tissue types, and lineage-specificity, based on data mostly in humans.

## 1. Overview of Transposable Elements and Their Role in the Human Genome

Transposable elements (TEs), also known as mobile elements (MEs), are interspersed repeats constituting a major fraction of the genomes in higher organisms. The contribution of TEs in the human genome has been updated to at least 50% using the recent versions of the reference genome sequence and TE annotations [1]. Based on the transposition mechanism, there are two classes of TEs: class I transposons, also called retrotransposons, that transpose by copy and paste mechanism, and class II transposons, also called DNA transposons, that transpose by cut and paste mechanism [2,3,4]. Class II TEs are less abundant in the human genome (3.5%) and are considered DNA fossils (remnants from the ancestral genome) as no family of DNA transposons currently remains active [5]. Retrotransposons, therefore, represent the major types of TEs in the human genome due to their replicative transposition and ongoing activity. There are different types of retrotransposons, including endogenous retroviruses (ERVs), which are characterized by the presence of long terminal repeats (LTRs) and non-LTR retrotransposons. Non-LTR retrotransposons are further divided into long interspersed elements (LINEs), short-interspersed elements (SINEs), and SVAs (chimera of SINE-R, variable number tandem repeats, and *Alu*-like, with SINE-R sequence, is derived from LTR sequence of HERV-K10). Non-LTR retrotransposons are characterized by polyA-tail and target site duplications (TSDs), with the former unique to this TE type but the latter common to all TEs [6,7]. LINEs have the largest contribution in the human genome at 20.4% followed by SINEs (13.1%), LTRs (9.1%), and SVAs (0.1%) [1,8]. SVAs are a very young and active class of TEs despite having only ~5000 copies in the human genome [9].

The previous notion of TEs being junk or selfish DNA has been revolutionized with the revelation of TEs’ role in genome evolution and gene function [10,11]. TE insertions tolerated during evolution have many effects on the structure and function of the human genome and, along with other genomic factors, shaped the evolution of human lineage [12]. The impact of TEs on human genome evolution has been thoroughly discussed in the earlier reviews by Ayarpadikannan and Kim [11] and Cordaux and Batzer [10]. To recapitulate, TEs are an important factor responsible for rearrangements in the human genome, including tandem duplications and insertion- and recombination-based deletions [13,14,15]. TEs are also involved in local genomic instability and have been found to generate microsatellites in the human genome [16,17]. Another impact of TEs is the creation of new genes with functions essential to the host [18,19]. These molecular domestication events repeatedly occurred during the evolution of eukaryotic lineages. One such well-established example is the retrotransposon-derived PEG-10 gene that is involved in placental formation [20,21]. Another important function of TEs in the human genome is their involvement in gene expression regulation. As will be discussed in this review, the two principal methods by which TEs regulate the expression of genes are the function as *cis*-acting regulatory sequences and encoding of regulatory RNAs. Ongoing TE insertions of certain TE subfamilies in the human genome can lead to insertions of TEs in genic regions and alteration in the level of gene expression via different mechanisms, including alternative splicing, the introduction of a premature stop codon, and introduction of polyadenylation and termination signals, etc. [22,23,24]. This can be considered as another way by which TEs can alter gene expression levels. Our review is, however, mainly focused on TEs’ direct participation in gene regulation via TE-derived *cis*-regulatory regions and TE-derived regulatory RNA sequences in the human genome. In this review, we aim to comprehensively cover the major studies regarding these two aspects of TE-mediated gene regulation in the human genome, and based on these studies’ findings to address questions including (1) What is the extent of TEs’ contribution and how versatile is the role of TEs? (2) Does TE-mediated gene regulation tend to be tissue-specific? Does TE-mediated gene regulation lead to evolutionary novelty? (3) How different classes of TEs differ in contributing to gene regulation?

## 2. *Cis*-Regulatory Activities of TEs

TEs considerably contribute to the *cis*-regulatory regions of the human genome. It has been observed that TEs contribute to almost half of the open chromatin regions [25]. Although accessibility does not equate to regulatory function, a recent review analyzing the relationship between physical and functional genome concludes that chromatin accessibility plays a wide role in defining active regulatory elements [26]. The fact that TEs contribute ~50% of the open chromatin regions demarcates the role of TEs in gene regulation. As established by different studies, TEs either provide alternative promoters and enhancers or alter the activity of existing promoters [27,28]. The jumping nature—along with the presence of intrinsic regulatory sequences in TEs for their own expression—as well as TEs’ susceptibility to recruiting silencing factors for their own suppression, make them a crucial player in controlling gene expression patterns. This section of the review will cover TEs’ *cis*-regulatory activities, including TEs’ involvement in important gene regulatory elements, genes that have been found to be controlled by TEs’ regulatory activities, spatial gene regulation by TE-derived *cis*-regulating elements, conservation of the TEs-derived *cis*-acting elements across species, and polymorphic TEs leading to population-specific gene expression patterns.

### 2.1. Contribution of TEs in Different Regulatory Elements in the Genome

#### 2.1.1. Regulatory Elements in the Genome

*Cis*-regulatory regions (including promoters, enhancers, silencers, and insulators) are non-coding DNA sequences that regulate gene expression by providing binding sites for *trans*-acting factors. Promoters are orientation-dependent regulatory elements with respect to the genes and provide a docking site for basic transcriptional machinery. Other regions that control transcription in the eukaryotic genome include enhancers, silencers and insulators. Unlike promoters, enhancers and silencers are orientation- and position-independent with respect to genes. Enhancers typically consist of clusters of transcription factor binding sites (TFBSs) that work cooperatively to upregulate gene expression. Silencers, in contrast, downregulate gene expression by recruiting factors that promote close chromatin structures. Insulators are another type of regulatory elements that protect genes from the regulatory influence of the surrounding genes. All of these regulatory regions in the genome play a crucial role in gene regulation by interacting with a wide range of *trans*-acting factors.

Databases of gene regulatory regions: To provide a comprehensive map of gene regulatory regions in the human genome, different approaches have been used, including identification of open chromatin regions, localization of binding sites of transcription factors (TFs) and other gene regulatory proteins and mapping of the chromatin states by identifying the sites of DNA methylation and active and repressive histone marks [29,30]. In order to acquire these datasets, a wide range of high-throughput functional genomics techniques have been utilized. For identification of open chromatin regions in the genome, the commonly employed DNA accessibility assays include DNase-seq, formaldehyde-assisted isolation of regulatory elements (FAIRE)-seq and assay for transposase accessibility and deep sequencing (ATAC)-seq [31,32,33]. For identification of TFBSs and binding sites of epigenetically modified histones, chromatin immunoprecipitation (ChIP)-seq technique is used [34]. For mapping of DNA methylation sites in the genome, Whole-genome bisulfite sequencing (WGBS) and reduced representation bisulfite sequencing (RRBS)—that only targets promoters/CpG islands) are the commonly employed assays [35]. There are different databases that provide gene regulation datasets by either reporting data of these experiments separately or by integrating the data of different assays to define promoter and enhancer elements in the genome. Two important databases providing the massive data of the functional genomics experiments mentioned above are the Encyclopedia of DNA Elements (ENCODE) project database [36] and Roadmap Epigenomics Mapping Consortium (REMC) project database [30]. These data encompass a wide range of tissues and cell lines. Some of the small-scale projects are Genomics of Gene Regulation (GGR), which includes data mainly for the A549 cell line and few primary cells, and the blueprint epigenome project database [37], which provides data for distinct types of hematopoietic cells. Based on these primary datasets, there are some secondary databases to provide a meaningful interpretation of the primary data in various ways. For example, an enhancer database, EnhancerAtlas [29], provides enhancer annotations across nine different species by combining the output of multiple high-throughput experiments. It integrates the ChIP-seq datasets of histone modifications, TFs, and other regulatory proteins that specifically bind to enhancers, different open-chromatin datasets (DNase-seq, FAIRE-seq, and MNase-seq), as well as the findings of some reporter assays to demarcate enhancer regions in the genome. Another enhancer database is SEdb [38], a comprehensive database of super-enhancers (large cluster of transcriptionally active enhancers) in the human genome. Table 1 summarizes the main primary and secondary gene regulation databases.

#### 2.1.2. Intrinsic Regulatory Properties of TEs

Many studies have revealed that TEs contribute to all regulatory regions described above [28,45,46,47]. Intrinsic regulatory properties of TE sequences make them suitable candidates for regulating gene expression. Like other genes, TEs may harbor the primary types of regulatory sequences for their own expression: promoters, enhancers/insulators, splice sites, and terminators. Internal regulatory sequences of the retroelements can be carried into the progeny copies [48,49]. LTRs and LINEs carry POL II promoters, while SINEs carry promoters for either POL III or POL II [48,50]. SVAs contain a core enhancer element [51] within the SINE-R sequence [52]. According to one of the models proposed for SVA transcription, the internal enhancer element of SVAs acts cooperatively with the external promoters to promote SVA transcription [53]. In addition to their regular internal promoters, some TEs, such as L1s, also contain active antisense promoters (ASP), which can drive the generation of additional antisense transcripts that usually extend into the neighboring regions to form chimeric transcripts of known genes as a mechanism to interfere normal gene expression [54].

#### 2.1.3. TEs Contribute to Regulatory Elements in the Genome

TEs’ exaptation to regulatory elements in the human genome has been well documented. “Exaptation” is a phenomenon in which a functional feature of phenotype was not a result of a natural adaption of the current role but co-opted structures for another function [55,56]. Here, we use the term specifically for referring to junk DNA sequences acquiring non-TE functions in the genome. TEs have been observed to originate conserved enhancer elements in the vertebrate genome [57]. Franchini et al. [28] discovered that an LTR retrotransposon (belonging to THE1B in the MaLR subfamily) exaptation causes the evolution of an enhancer element, which leads to neuronal-specific expression of the *POMC* gene in mammals. LTR retroelements of this subfamily have also been found to be involved in abnormal expression of the *CSF1R* gene in Hodgkin lymphoma. In this case, transcription of *CSF1R* in transformed human cells was found to be initiated at an anomaly activated LTR retroelement [58]. Another study showed that the insertion of an ERV repeat in the upstream region of the *AMY1* gene leads to the activation of cryptic promoters and tissue-specific expression of the gene [45]. Moreover, in the study by Wang et al. [59] and Lu et al. [60], HERV-H retroelements were shown to act as enhancers and drive the expression of pluripotency-modulating lncRNAs in human embryonic stem cells (hESCs). The studies showed that disruption of HERVH and HERVH-derived transcripts is linked to morphological changes and reduced pluripotency in the cells. Two reports established the role of *Alu* elements in the evolution of T cell promoters and enhancers: an *Alu*Sp in the promoter of *FCER1G* gene induces T cell expression; an *Alu*Y in the intron of human *CD8* gene acts as a T cell enhancer. Both these *Alu* sequences harbor the binding motifs of Lyf-1 TF, which drives T cell-specific expression [46,47]. Transcription of the *Alu*Sq from its POL III promoter prevents the human epsilon globin gene from regulation by the activities of the other upstream promoters, showing *Alu* as an insulator [61]. A study by Kim and Hahn [62] identified alternative promoters derived from L1 and SVA elements in *CHRM3* and *WDR66* genes, respectively [62].

It is worth mentioning here that not all studies investigating TEs in the regulatory elements are conclusive about TEs’ role in gene regulation. First, there is controversy regarding the functional significance of genes’ transcripts induced by TE-derived alternative promoters. For example, the study of Kim & Hahn [62] mentioned above identified transcript variants induced by TE-derived alternative promoters. However, as reviewed in Cohen et al. [63], these can be minor mRNA variants with no functional significance. Nevertheless, the study by Lamprecht et al. [58] determined CSF1R gene transcription driven by LTR-derived alternative promoter in human lymphoma cells with the functional significance of the mRNA variant verified by relating mRNA and protein expression data and by showing inactivity of canonical promoters in these cells. Second, detecting the biological significance of TE sequences (e.g., binding to TFs) in promoters and enhancers, and even affirmation of transcriptional activity of these TEs is not entirely incontrovertible regarding TEs’ significant role in gene expression (as reviewed in de Souza et al. [64]). Many of the studies mentioned in the previous paragraph have experimentally confirmed the transcriptional activity of TE-derived sequences in regulatory elements [28,46,57]; however, there are not many next level studies for determining the physiological and morphological changes caused by these TE-derived sequences in the human genome.

Besides experimental studies, recently, the contribution of TEs in the promoters of genes expressed by POL II was determined using ENCODE and RepeatMasker annotations for TFBSs and TEs, respectively, by analyzing promoters as the 1500 bp regions upstream of the transcription start sites (TSSs). Out of the 35,007 promoters, 75% were found to have TE-derived sequences, with some promoters found to have as many as ten TEs [65]. Although the study showed that a large fraction of promoters has TE sequences, this finding is not very convincing regarding TEs’ role in gene regulation. The study observed only 6.8% of the TFBSs in promoters to be TE-derived. Moreover, the study by Simonti et al. [66] showed contrasting findings. They analyzed the promoters within 1 Kb of annotated TSSs identified by the Functional Annotation of the Mammalian Genome (FANTOM) consortium for TE enrichment and determined that promoters are significantly depleted of TEs.

In a recent work by Zeng et al. [67], TE enrichment was determined in different regulatory regions by measuring “P(TE|RE)”, the probability of nucleotide in the regulatory element being from the TE. Interestingly, P(TE|RE) was found to be higher in repressors than promoters, reaching 0.2 and 0.5 for promoters and repressors, respectively [67]. The role of TEs as gene repressors has also been supported in other studies showing that TEs can repress nearby genes by spreading local heterochromatin [68,69]. The study by Brattås et al. investigating the ERV expression pattern in the human brain revealed that TRIM28, a corepressor protein, binds on the docking site on ERV and consequently regulates the nearby genes [69]. L1-mediated transcriptional repression of neighboring genes also has been observed in human cell lines [68].

In summary, studies have revealed TE sequences embedded in regulatory elements, as well as the regulatory role of these TEs. Besides their contribution to canonical promoters, TEs have also been found to create alternative promoters for certain genes. From the studies mentioned in this section, it can be concluded that TEs are the reservoir of diverse regulatory functions and play an important role in the evolution of different types of regulatory elements.

#### 2.1.4. Contribution of TEs to TFBSs

Studies have documented the binding of TFs to TEs and showed TEs have TF-binding sequence motifs [65,70,71]. TE sequences widespread in the human genome could provide binding sites for many classes of TFs [65]. As examples, a large fraction of binding sites for ESR1, TP53, POU5F1, SOX2, and CTCF are embedded in different TE families [72]; MER41 retrotransposons harbor binding sites for STAT1 [73]; the binding sites for four TFs (ERα, FoxA1, GATA3 and AP2γ) act as a regulator of mammary gland development arose from the spread of TEs [74]. In the study by Sundaram et al. [70], TF binding regions (TF ChIP-seq binding peaks) of 26 TFs were analyzed in two human cell lines (K562 and GM12878), and it was observed that 20% of the TF binding peaks belonging to a wide range of TFs were found to be derived from TEs [70]. TEs contribute to TFBSs by providing ready-to-use TFBSs immediately after insertion and by generating novel TFBSs via post-insertion random mutations. The presence of TF-binding motifs in TEs prior to their insertion has been indicated in work conducted by Ito et al. [75]. The study determined TFBSs in the LTR retroelement (HERV-TFBSs) and later determined TF-binding motifs that were found in a substantial fraction of HERV-TFBSs at the same consensus position (named ‘HERV/LTR-shared regulatory element—HSRE’ by the author). HSREs were found in 2% of all the TFBSs in the genome [75]. In addition to the use of existing TFBSs, the creation of TFBSs in TEs after their insertion also has been reported. For example, methylated CpGs of human *Alu* sequences can undergo deamination (C->T mutation) to create a binding site for c-Myc TF [76]. Another study revealed that a single C to T substitution in the *Alu* sequence leads to a functional binding site for Lyf-1 TF [47]. Deamination of CpG in *Alu* sequences also has been found to originate binding sites for RAR [77]. Likewise, deamination of methylated CpG sequences to TpG in human LTRs has been shown to create binding sites for p53 [76]. The role of mutations in TEs in providing new regulatory sequences is supported by genome-wide studies analyzing TE-derived TSSs in the human transcript libraries, which showed that old L2 elements are more likely to contribute to promoters than new L1s [78].

The occurrence of TFBSs across TEs in the human genome is not random. Binding sites of a TF are enriched in copies of specific TE families. A total of 710 such TF-TE relationships have been identified [70]. Nonrandom association of TEs with TFBSs is also indicated by TEs providing combinatorial interaction of TFs. TEs provide clusters of binding sites for TFs that work cooperatively in gene regulation. For example, the MIR family of SINEs that have an affinity for estrogen receptor α (ERα) also provides binding sites for ERα co-factors [79]. The nonrandom association of TEs with TFBSs signifies the role of TEs in shaping gene regulation networks.

TEs are considered as a source for a large number of TFBSs in the human genome. It has been observed that TFs with a greater number of TF ChIP-seq peaks not only have a greater number of TE-derived peaks but also have a greater fraction of TE-derived peaks indicating TEs being responsible for generating certain TFBSs [70]. Another study analyzing the role of genome expansion in the evolution of gene regulation indicates that TFs increase their targets in the genome through genome expansion, mainly by repeat elements [80]. The study determined the age of human genomic regions and their TFBS distribution by applying the parsimony model to the genome-wide alignment of 100 vertebrates. It was found that binding sites of a TF were enriched in genomic regions of a given age, suggesting that new genomic sequences provide new targets for existing TFs [80]. In concordance with the role of TEs in expanding TFBSs, TE-derived TFBSs are considered as the marker of gene regulation evolution. In the study by Nikitin et al. [81], the evolution of transcriptional regulation was determined for different genes and pathways using retroelement-derived TFBS as a metric. Genes enriched for TE-derived TFBSs and the associated pathways were considered to have high evolutionary rates.

The functional significance of TE-derived TFBSs in the human genome has been highlighted in several folds. First, functionally important positions of TE-derived TFBSs that interact with TFs are more conserved than adjacent positions as a sign of functional constraints on these TFBSs [82]. Second, TEs that are de-repressed in cancers have been found to harbor binding sites for oncogenic TFs, including C/EBPβ, E2F1, and MYC [83]. In the study by Kellner and Makałowski [65], 6.8% of TFBSs present in the promoters were found to be derived from TEs, indicating their regulatory function. Moreover, TE sequences are not associated with genes, but harboring TF binding motifs could participate in gene regulation by acting as competitors of the genes’ regulatory sequences in binding to TFs.

#### 2.1.5. Differential Contribution of TEs by Type in Regulatory Regions

The contribution of TEs to the regulatory elements in the human genome varies among TE types. The study by Zeng et al. [67] determined the proportion of nucleotides belonging to different types of TEs in regulatory regions. It revealed that *Alu* elements contribute most to all types of regulatory regions, while L1s were found to be least likely in the regulatory regions. The authors of the study reasoned that the large size of L1s and even truncated L1 copies might disrupt the genic regions of the genome, and therefore L1 insertions in the regulatory elements have not been evolutionarily favored. Furthermore, as L1s on average are older than *Alu* elements, a more significant contribution of *Alu* elements than L1s in different types of regulatory elements was considered as indicative of the idea that clade-specific and species-specific TEs are more likely to contribute to gene regulation. This finding is also supported by the study of Nikitin et al. [84], which revealed that SINE-derived TFBSs are more in number than LINE-derived TFBSs in gene neighboring regions (5 Kb surrounding TSS), while it is the opposite for regions outside the gene neighborhood. Another support has been provided by the recent study by Kellner and Makalowski [65], which indicated that SINEs are more frequent in promoters (1.5 Kb upstream of TSS) than non-promoter regions, while it is the opposite for LINEs. Hence, multiple studies have shown in different ways that SINEs might contribute more to regulatory regions than LINEs. However, it should be noted here that these computational studies are based on sequence analysis, which is prone to noise and methodological biases. Therefore, it is critical that these data, for example, the biological function of the SINE-derived TFBSs in gene neighboring regions, are subject to experimental verification.

Although the presence of *Alu* elements in regulatory elements signifies the role of lineage-specific TEs in gene regulation, it has been found that ancient repeat elements, including L2 and MIRs, show a higher nucleotide proportion in enhancers despite having lower sequence contribution to the genome [67]. In another study, analysis of TE-derived TFBSs showed that ancient TE families like MIRs and L2s are more enriched for TE-derived TFBSs than younger families like *Alu* elements and L1s [82]. As suggested by the authors, the presence of ancient TEs in these TFBSs highlights the functional conservation of TE-originated regulatory sites [82]. Based on these findings, it can be said that although the exaptation of younger TEs to regulatory elements evolves gene regulation, certain classes of regulatory elements are enriched for older TE families indicating functional conservation of TE-originated regulatory sites.

Besides SINEs and LINEs, LTRs are also considered as an important TE class in gene regulation as they retain their regulatory sequences after their integrations, and they are the most dominant TE class in open chromatin regions of the human genome [25]. Moreover, ERVs/LTRs are the most diverse class of human TEs, providing various regulatory elements and TFBSs [73,75]. The study by Thornburg et al. also showed that unlike LINEs, SINEs and DNA elements, LTRs are enriched for binding sites of the majority of TF classes [85]. Investigating the regulatory properties of different classes of LTRs has therefore remained an important area in TE-mediated gene regulation. However, as mentioned earlier, studies analyzing the number of TE-derived TFBSs for different types of TEs in upstream gene regions have not found the major contribution of LTRs, which implies that LTRs may be involved in regulating distant genes.

In summary, we reviewed in this section TEs’ contribution to the major regulatory elements in the human genome, highlighting some important functional aspects of TE-mediated gene regulation like activation of cryptic promoters by TEs and combinatorial interactions of TFs contributed by TEs. The role of TEs has been observed in promoters, enhancers, and silencers. This diversity of TE-mediated gene regulation can be linked to a wide variety of TFBSs provided by TEs and different types of intrinsic regulatory properties present in TEs for their own regulation. Nevertheless, studies involving experimental verification of the functional role of TEs in regulatory elements are still limited, and future work in this direction can employ methods such as reporter gene expression under the control of promoters with and without the TE-derived sequences to elucidate TEs’ specific roles in gene regulation.

### 2.2. Genes Regulated by TE-Derived cis-Regulatory Sequences

Many genes in the human genome have their expression known to be controlled by TE-derived regulatory sequences. Some studies focusing on specific genes have identified TE-derived regulatory elements by using a reporter gene expression approach or by identifying alternative transcripts initiated at TE sequences. A few of these studies were already highlighted in the previous sections, and as examples, *POMC*, *CSF1R*, *FCER1G*, and *CD8* genes are regulated by TE-derived regulatory elements [28,45,46,47,58].

Genome-wide analysis has also been conducted by different research groups to identify TEs in the gene upstream regulatory elements. The study by Kellner et al. [65] showed that 75% of the 35,007 genes transcribed by POL II have TE-derived sequences in their promoter regions, which represents enrichment over the genome average. This coincides with the TEs’ preferential insertion in the upstream gene regions [86]. The same study further identified that for two protein-coding genes, *PCBD1* and *PPP1R3A*, almost all the entire promoters are derived from TE sequences [65]. The study by Nikitin et al. [84] showed that among the protein-coding genes, *USP176L26*, *USP17L13*, and *USP17L12* genes (encoding ubiquitin associated peptidase) most strongly associate with TE-derived TFBSs.

TEs can also regulate the far away genes by acting as enhancer elements. Raviram et al. [87] analyzed 3D genomic interactions to determine the genes regulated by ERVs. They used Chromosome Conformation Capture (3C) methodologies to determine the transposons’ contribution to chromatin folding and long-range intra-chromosomal interaction and provided a strategy to identify TE-regulated genes, specifically genes interacting with TE-derived enhancers. It was found that the *IF16* gene is upregulated by a retroelement MER41B. The gene’s promoter was found to be interacting with this LTR located ~20 Kb downstream of the gene. Similarly, the technique captured the interaction between *IFITM* (*IFITM1* and *IFITM3*) genes and MER41A retrotransposons located downstream of the genes. Expression of the *MYPN* gene was also found to be regulated by distant TE enhancers [87]. The long-range gene regulation by TEs has also been indicated in the study by Zhang et al. [88]. They showed that HERV-H defines the boundaries of topologically associated domains (TADs) in human pluripotent stem cell (hPSC), and its deletion eliminates the boundaries and reduces the expression of genes in the domain. All these examples signify the importance of unveiling the long-range genomic interaction of TEs in identifying TE-regulated genes.

In summary, the expression of a certain number of genes has been experimentally validated to be controlled by TEs, followed by recent genome-wide data analytical studies that have revealed TE sequences in many gene regulatory regions underscoring the need to further investigate the topic. Genes with TE-derived regulatory sites have a wide range of functions, with their products including neuropeptides (*POMC*), muscle protein (*MYPN*), immune receptors (*FCER1G* and *CD8*), metabolic enzymes (*AMY1*), and signaling receptors (*CSF1R*), and many others. The functional diversity of the genes being regulated by TEs indicates TEs’ diverse impact on host phenotype. Further, as to be discussed in detail later, some studies also showed that genes crucial for speciation novelty have TEs in their regulatory regions, highlighting the importance of TEs in evolution and functional diversity.

### 2.3. Tissue-Specific Gene Regulation by TEs

The epigenetic status of TEs varies across human tissues [89], leading to the varying profile of TE regulatory activities in different tissue types. Tissue-specificity is considered as one of the ways in which TEs contribute to evolutionary novelty in gene regulation. Studies focusing on specific genes have revealed TEs’ exaptation to tissue-specific regulatory sequences. For example, as mentioned before, an LTR retroelement provides neuronal enhancer of *POMC* gene and immune genes, and *Alu* sequences were found to provide T cell promoter and enhancers for *FCER1* gene and *CD8* gene, respectively [28,46,47,73].

Genes with LTR retroelement in the upstream regions have been found to exhibit tissue-specific expression compared to LTR-unassociated genes [90]. This systematic study analyzed gene expression data of 18 different tissue types from Illumina Human Body Map 2.0 (HBM2.0), and determined co-expression of LTR-associated and LTR-unassociated genes, and found 62 LTR elements linked to tissue-specific gene expression [90]. Trizzino et al. [91] used the data of the “roadmap epigenomics project” and “genotype tissue expression project” to determine TE presence in active and repressed chromatin of different tissues and the consequences on the gene expression. Interestingly, genes having the same expression in different tissues (i.e., lack of tissue-specific expression) rarely have TE insertions in their regulatory regions. It was found that TEs’ (particularly LTRs) involvement in the active chromatin regions varies across tissues. For instance, HERV15 is significantly enriched in active chromatin of liver tissue, while X7C (LINE) and Charlie15a (DNA transposon) are enriched in the active chromatin of breast tissue. Further, the tissue-specific TE involvement in active chromatin was linked to tissue-specific gene expression. It was revealed that TEs in the active chromatin regions of tissues have binding sites for that tissue’s key TFs. For example, HERV15 is more enriched in the active chromatin regions of the liver, and it has binding sites for EOMES, a key TF in the hepatic immune response. The tissue-specific involvement of TEs in active chromatin regions was also found to be associated with altered gene expression levels in that tissue [91]. The study by Kellner and Makalowski [65] examined the ENCODE data of TFBSs in six different tissues (blood, breasts, kidney, liver, lung, and stem-cells) in a pairwise fashion and found that only a small fraction of TE-derived TFBSs active in one tissue was used in another tissue. For example, only 3% of TE-derived TFBS active in blood tissue was also used in breast tissue. For almost all the tissue pairs, this percentage was significantly smaller for TE-derived TFBSs than for all TFBSs, indicating the role of TEs in tissue specificity of gene expression. As an example, 9% of all TFBSs active in blood tissue was also active in breast tissue, but just 3% of the TE-derived TFBSs active in blood tissue were also used in breast tissue [65]. Moreover, a very recent study analyzing ENCODE data for human GM12878 and K562 cell lines showed that variability in the TE-derived CTCF sites across different cell types leads to chromatin looping variation and alternative promoter-enhancer interactions associated with the difference in gene expression across cell types [92].

As highlighted by the studies mentioned above, the tissue-specificity of TE-mediated gene regulation has been corroborated using different approaches. Many TEs providing *cis*-regulatory sequences tend to function in a tissue-specific fashion and play an essential role in the differential gene expression across tissues.

### 2.4. Lineage-Specific Gene Regulation by TEs

**Figure 1:**
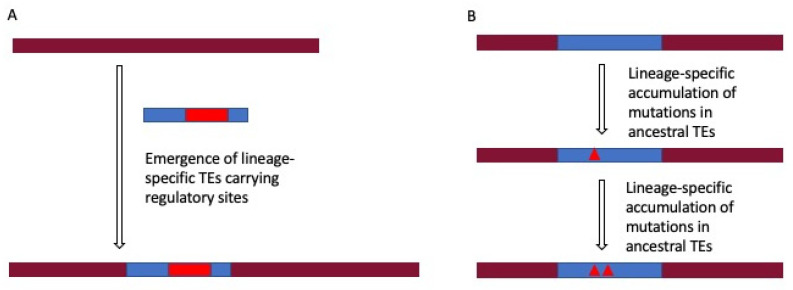
Two different pathways of generating lineage-specific transposable elements (TE)-derived regulatory sites. Lineage-specific TE-derived regulatory sites arise due to the emergence of lineage-specific TEs in the genome (**A**), or it may be due to the accumulation of mutations in ancestral TEs in a lineage-specific fashion (**B**).

TEs have been observed in the lineage- and species-specific regulatory regions implying the role of TEs in evolving gene regulation. The study by Rayan et al. [77] revealed that 56% of the anthropoid-specific regulatory elements have a TE origin, while Trizzino et al. [93] compared human liver promoter and enhancer sequences across six primate species and found that the majority of the non-conserved regulatory elements are enriched for TEs including LTRs and SVAs [93] with SVAs being hominid-specific [9]. The emergence of TE-derived lineage-specific regulatory sites is either due to newly evolved lineage-specific TEs or might be due to lineage-specific mutations in the ancestral TEs [78,94,95] (Figure 1). The creation of gene regulatory sites by mutations in the ancestral TE sequences is supported by the finding that most of the TEs in the regulatory regions have a high sequence divergence (>8% diverged) [84]. This has also been considered as the reason behind the higher contribution of ancestral TE families (L2 and MIR) than that of L1 and *Alu* in some regulatory regions, as mentioned before in Section 2.1.4 discussing the generation of new TFBSs in the genome by mutations in TE sequences. Moreover, lineage-specific TEs are also the source of lineage-specific TE-derived regulatory sites. Different vertebrate lineages contain quantitatively and qualitatively different populations of TEs, essentially due to different evolution of ancestral families of TEs, the lineage-specific introduction of TEs by infection, and lineage-specific emergence of new TEs subfamilies, as well as an ongoing transposition from existing active TEs. Lineage-specific TEs have been revealed to participate in lineage-specific gene regulatory regions. In a recent study by Pontis et al. [96], evolutionarily young and hominid specific TEs belonging to LTR5Hs/HERVK, LTR7/HERVH, and SVA subgroups were found to act as enhancers in human embryonic stem cells (hESCs). Another study showed that only 5% of TFBSs for *Oct4* and *Nanog* (key regulators of embryonic stem cells) are conserved between human and mouse embryonic stem cells, and the majority of the non-conserved sites reside within species-specific LTRs [95]. This links the emergence of species-specific TEs to the evolution of gene regulatory networks involved in pluripotency and cell fate determination. Another study indicates the role of transposons in gene regulatory networks crucial for speciation novelty (e.g., pregnancy in eutherian mammals). It was found that 13% of the genes showing endometrial expression in placental mammals had eutherian-specific TEs in the upstream region [94]. Moreover, it has been found that in the human genome, 30% of the TFBSs of the tumor suppressor protein, p53, reside in the primate-specific ERV regions [97]. The findings of these studies show that the emergence of species/lineage-specific TEs contributes to the evolution of gene regulatory networks pertinent to significant biological functions, including pluripotency of ESCs, lineage-specific traits like pregnancy in placental mammals and tumor suppression.

The higher contribution of ancestral TE subfamilies (L2 and MIR) than L1s and *Alu* elements in some regulatory regions might seem contradictory to the lineage-specificity of TE-mediated gene regulation. However, as mentioned before, sequence divergence of ancestral TEs evolves regulatory regions in species. Nevertheless, TEs indeed have also been identified in the conserved mammalian-wide regulatory elements, for example, a neuronal-specific TE-derived enhancer of the *POMC* gene exapted before the origin of prototherians (~166 Mya) [28]. Concludingly, besides providing conserved regulatory functions, TE-derived regulatory sites also tend to be species/lineage-specific and contribute to speciation novelty and diversity. Future comprehensive analysis encompassing all categories of regulatory elements across a wide range of species should provide more insight.

### 2.5. Population-Specific Gene Regulation by Polymorphic TEs

The majority of the TEs in the human genome are fixed and derived from ancient transposition events, and previous studies exploring the regulatory effects of TEs mostly have focused on the ones fixed in the human population. Nevertheless, mobile element insertion (MEI) polymorphisms have been found to be the most frequent structural variants in the human genome. The three families of retrotransposons primarily responsible for generating human TE polymorphisms are *Alu* elements, L1s, and SVAs [9,98,99,100]. LTRs, despite having presently limited activity, also account for polymorphic TEs in the human population [101], and there are studies reporting HERV-K insertion polymorphisms [102,103].

It is estimated that, on average, the two haploid human genomes in the same individual differ by about 1000 TEs insertions [104]. More than 16,000 polymorphic TE loci were identified in the recent phase 3 variant release of the 1000 Genome Project [98]. Furthermore, a recent analysis of deeply sequenced whole-genome data of 152 populations from “The Simon Genome Diversity Project” discovered more than 5000 additional MEIs not reported by the 1 K genome project [105]. Based on TEs’ intrinsic regulatory activity, it is very likely that polymorphic TEs are involved in differential gene expression among human populations by offering new regulatory sites to their nearby genes. The presence of such MEIs in the population is likely subject to selection, while in some cases, their impact on gene regulation may contribute to disease, in addition to the well-documented disease causing mostly by interrupting normal splicing and/or open reading frames (see recent review by Kazazian and Moran [106]).

Limited studies have shown that many polymorphic TE loci in humans correspond to *cis*- and *trans*-eQTLs [107,108]. The study by Wang et al. [108] investigated the association between polymorphic TE loci and gene expression level. In the study, genotype calls for polymorphic TEs were taken from the phase 3 variant release of the 1000 Genomes Project, and corresponding RNA-seq data for the same 1000 Genome Project samples were retrieved from the GUEVADIS RNA-seq project [109]. It was found that polymorphic TE loci were associated with differences in expression between European and African population groups. A single polymorphic TE locus was indirectly associated with the expression of numerous genes via the regulation of the B cell-specific TF [108]. In a recent extension of this work [107], rare and less common TE structural variant (TEV) polymorphisms (MAF < 5%) were also included, and a total of 323 significant TEV-*cis*-eQTL associations were identified.

Hence, far, there have not been many studies relating human polymorphic TEs with gene expression differences among populations. The work is limited to only five populations of the 1000 Genome Project data, as only for these populations, the corresponding RNA-seq data are available. Moreover, only lymphoblastoid cell gene expression level has been analyzed in these samples. There is a need for more detailed studies encompassing different tissue types and better population coverage to investigate further the correlation between polymorphic TEs and population or even individual level gene expression differences.

## 3. TEs Contribute to Non-Coding Regulatory RNAs

Advancement in RNA-seq technologies has dramatically increased the discovery of new RNAs, the ncRNAs in particular [110,111,112]. The wealth of ncRNAs is indicated by the fact that about 75–85% of the human genome gets transcribed despite only ~1.2% of the genome encoding proteins [113]. ncRNAs include housekeeping RNAs (rRNA, tRNA, snRNA, and snoRNA) and regulatory RNAs (small non-coding RNA (sncRNA) and long non-coding RNA (lncRNA)). Examples of sncRNAs are miRNAs and piRNA. miRNA plays an important role in gene regulation by interacting with the complementary sequence on the 3’ UTR of target mRNA, which leads to the cleavage or translation repression of the target mRNA. lncRNAs are further classified based on the genomic region they get transcribed: 1. LincRNAs transcribed from the intergenic regions; 2. Intronic lncRNAs transcribed from introns; 3. lncRNAs that are antisense transcripts of coding regions but do not encode proteins; 4. Circular lncRNAs that have scrambled exon sequences (due to exon shuffling) but do not encode proteins. A plethora of lnc/sncRNA genes has been identified. A total of 15,941 lncRNA and 9882 sncRNA genes have been documented in Gencode v24 [114].

snc/lncRNAs participate in a wide range of regulatory functions by either inducing degradation of mRNA transcripts or regulating the transcription. There is a close association of TEs with regulatory RNAs, as a significant number of these ncRNAs have originated from TEs. This section of the review will highlight TEs’ contribution to the regulatory RNAs, mainly focusing on the role of TEs in the origin, functionality, and diversification of regulatory RNAs.

### 3.1. Contribution of TEs to the Makeup of Regulatory RNAs

miRNAs are transcribed from genes as primary miRNAs (pri-miRNAs), which are further processed to precursor miRNAs (pre-miRNAs). These initial forms of miRNAs have a stem-loop structure, which is later cleaved to form mature miRNA, which is further loaded on Argonaute protein to perform gene silencing function [115,116]. Studies have reported the involvement of TEs in the origin of human miRNAs, particularly the stem-loop structure of different miRNAs families. Supported by the TE-origin of many miRNAs, it has been hypothesized that the presence of two similar TEs flanking a genomic locus leads to the formation of miRNA stem-loop structure [117]. Another study reported an observation of high sequence identity between the miRNAs of the hsa-mir-548 family and the miniature inverted-repeat transposable elements (MITEs). MITEs form a stem-loop structure, which can be recognized by RNAi enzymes and processed into mature miRNA [118]. In the study by Yuan and colleagues [119], it was shown that the MER53 elements, a subclass of TEs characterized by the presence of terminal inverted repeats (TIRs) and TA target site duplications that can form palindromic structures, gave rise to all members of the miR-1302 gene family [119]. In another study, analysis of human palindromic MER sequences using miPred (a tool that distinguishes real miRNA precursor from other hairpin sequences) identified three miRNAs derived from a MER96 located on chromosome 3 and MER91C paralogs located on chromosome 8 and chromosome 17 [120].

TEs have been found to have overlap with pre-miRNA sequences as well as in mature miRNAs. Small RNA sequencing coupled to argonaute2 RNA immunoprecipitation (that captures mature miRNAs) has identified TE-derived miRNA sequences. In a recent study by Petri et al. [121], TE-derived miRNAs in human brain tissues were identified by conducting Argonaute2 RNA immunoprecipitation followed by small RNA sequencing (AGO2 RIP-seq). The study determined a total of 19 miRNAs that were derived from L2. It was speculated by the authors that these L2-miRNAs could target many protein-coding genes carrying L2 sequences in their 3’ UTRs [121]. Many bioinformatics studies are highlighting the overlap of TEs with miRNA genes. miRBase is a publicly available online repository for miRNA sequences and annotations, allowing researchers to examine the contribution of TEs to miRNA sequences. In the study by Piriyapongsa et al. [122], 462 human miRNA gene sequences from the miRbase database were analyzed, and 68 were shown to contain TE sequences. Further, a negative correlation was observed between the expression level of TE-derived miRNAs and their putative target genes [122]. In another study, miRBase data were analyzed to detect repeat-derived miRNA (Rdmir) in different species, in which a miRNA was defined as a Rdmir if at least 50% of it overlapped with TE sequences. Using this rule, a total of 226 miRNA genes were identified in humans as Rdmirs [123]. Analysis of 6845 pre-miRNAs from eight different vertebrate species in the study by Qin et al. [124] showed that miRNAs derived from TEs (MDTEs) account for 19.8% of miRNAs in the human genome, which include a total of 409 TE-derived miRNAs (386 overlapped with TEs and 23 un-overlapped with TEs). The proportion was higher than those of other vertebrates. MDTEs with un-overlapped TEs are those miRNAs that are derived from TEs but losing their TE sequences during evolution. Such MDTEs were determined by analyzing miRNAs un-overlapped with TEs and comparing them with homologs in other vertebrates. After excluding multi-copy MDTEs, 338 unique MDTEs (UMDTEs) were identified. These UMDTEs were further classified into type I UMDTEs derived from inverted TE sequences (11.24%), type II UMDTEs with sequences partly overlapping with TE sequences that were not inverted (51.78%), and type III UMDTEs with sequences entirely derived from TE sequences (36.98%) [124]. A database named MicroRNAs Derived from Transposable Elements (MDTE DB) catalogs all the MDTEs identified by computational analysis of pre-miRNA sequences in miRbase (v20). The database reports 2853 MDTEs. In humans, about 250 partially covered and 150 wholly covered MDTEs have been identified [125]. It is worth noting that these studies analyzed miRNA sequences from earlier versions of miRbase. The miRbase archive of miRNA sequences has been increasing quickly and the latest version miRBase (v22) released in 2018 reports 48,860 mature microRNAs from 271 organisms [126]. There are more than 20,000 new entries in this version and the sequence has been changed for more than 800 entries. This demands the latest update of MDTEs based on the current version of miRbase.

As for miRNAs, the contribution of TEs in human lncRNAs has also been established by several studies. For example, a study analyzed 19,835 lncRNA transcripts from Gencode v13 and found that 75% of these lncRNAs transcripts have TE sequences [127]. In another study, 61 of the 94 human lncRNA transcripts (65%) in the lncRNA database (lncRNAdb) were shown to have embedded TEs, making 27% of these lncRNA transcripts length. lncRNA genes harboring TEs were enriched in human chromosome 11, while chromosomes 16, 17, and 21 lacked lncRNAs containing TEs [128]. With consistent growth, the recent release of Gencode (v34, April 2020) catalogs 17,960 lncRNA genes and 270,000 transcripts [129], justifying an updated study regarding TE-derived sequences in lncRNA genes. Moreover, because of differences in the definitions of what constitutes lncRNA, the number of lncRNAs in the human genome drastically varies across different databases, including Gencode [130], FANTOM CAT [131], NONCODE [132], among others. To address this issue, large-scale annotations combining all lncRNA databases into one compendium are provided by the European Bioinformatics Institute (EMBL-EBI) comprehensive database RNACentral [133]. Another highly consistent database is LNCipedia that also provides functional annotations of lncRNA genes by an extensive manual literature curation, currently containing 1555 functionally annotated lncRNA genes [134]. Analyzing these all-inclusive lncRNA datasets and functionally annotated lncRNAs for embedded TE sequences should provide a rational extension to the existing studies.

Many lncRNAs are transcribed from intergenic regions (lincRNAs) and play a crucial role in gene regulation. lincRNAs constitute most of the lncRNAs, and they are considered as the largest class of ncRNAs in the human genome with >8000 lincRNA genes defined [135]. Thus, there have been studies explicitly focusing on lincRNAs. The study by Kelly and Rinn [136] provided a comprehensive analysis of human TE sequences in lincRNAs by obtaining RNA-seq data for 28 different tissues and cell lines. It was found that 7700 lincRNAs overlapped with TEs, and 1530 lincRNAs were depleted of TEs, indicating 80% of lincRNA genes associated with TEs and TEs comprise 42% of the total lncRNA sequences [136]. In work by Kannan et al. [137], 69% of 589 human lincRNAs from the NRED database were found to have TE-derived sequences. Further, different regions of human lincRNA genes were analyzed for the contribution of TEs. The percentage of TE-derived sequences in lincRNA genes was the highest for introns (>45%), followed by exons (>20%) and promoters (>10%). The distribution was similar to that of protein-coding genes. However, the content of TEs in lincRNA genes was substantially higher than that in protein-coding genes, especially in exons and promoter regions, which is indicative of the low functional constraints for lncRNA genes [137].

TEs have, therefore, clearly made a significant contribution to regulatory RNAs (miRNAs and lncRNAs). Palindromic sequences of certain TE families play crucial roles in the hairpin structure of miRNAs, and different TEs are linked to different miRNA families. TE sequences have also been found in non-hairpin mature miRNAs. The presence of TEs in all regions of lncRNA genes (promoters, introns, and exons) highlights TEs’ contribution to the generation of lncRNAs.

### 3.2. Functional Significance of TEs in Regulatory RNA Sequences

TE-derived sequences also impart functional properties to different types of sncRNAs and lncRNAs, making them essential for regulatory RNA functions, as demonstrated by the studies described below.

First, the TE-derived sequences have crucial roles in different types of human sncRNAs. miRNAs harboring TE sequences have been found to target genes with embedded TE sequences in 3’ UTR. For example, LINE2-derived miR-28-5p and miR-151 target Ly6/Plaur domain-containing 3 (*LYPD3*) and ATP synthase mitochondrial F1 complex assembly factor 1 (*ATPAF1*) genes, respectively, through pairing to LINE2 elements on 3’ UTR [138]. The subsequent study showed that miR-28-5p also regulates the expression of *LYPD3* and E2F transcription factor 6 (*E2F6*) genes through 3’ UTR harboring LINE2 sequences [139].

Second, TEs have also been found to have a diverse role in human lncRNA functions. *Alu* sequences are involved in the base pairing of lncRNA to its target mRNA, which is required for decaying target mRNA. In such cases, *Alu* sequences are present on both lncRNA and mRNA, which can lead to the formation of short imperfect pairing between the two RNA molecules. For example, a 3’ UTR *Alu* element of the plasminogen activator inhibitor type 1 (*SERPINE1*) gene binds to lncRNA harboring *Alu* sequences. The dsRNA structure is further degraded through staufeb1-mediated decay [140]. *Alu* elements have also been proposed to be involved in the circularization of circular lncRNAs. Circular lncRNAs make an important class of regulatory RNAs and impact gene regulation by influencing the transcription, mRNA turnover, and translation. They harbor exons out of order from the genomic context and are generated by exon shuffling via non-co-linear splicing. *Alu* sequences in introns flanking the exons are thought to produce circularization through *Alu*/*Alu* base pairing [141]. TEs also provide preformed structural and sequence features to lncRNAs, which imparts them the ability to interact with other biological molecules, including DNA, RNA, and protein. The repeat insertion domain of lncRNA (RIDL) hypothesis was proposed based on the concept that TEs serve as the functional domain of lncRNA [142]. For example, the ERVB5 sequence on XIST lncRNA provides binding sites for polycomb repressive complex 2 (PRC2) that contributes to chromatin compaction [18]. TEs have a significant influence on the lncRNA gene structure, and it has been found that TE-derived sites are present in promoters, splice donors, splice acceptors, and polyadenylation sites of lncRNA genes [127]. In a study by Kelley and Rinn [136], 127 lncRNAs were found to be upregulated by a HERV-H element acting as promoters of these lncRNAs. Based on this observation, it was proposed that TEs, such as HERV-H, can give rise to new lncRNAs by inserting active promoters into previously inactive genomic regions [136]. TEs have also been proposed to assist lncRNA in the formation of stable secondary structures. To assess this hypothesis, a study retrieved lncRNA data from GENCODE and compared lncRNAs with TEs to lncRNAs without TEs. Comparing the minimum free energy (MFE) of predicted secondary structures using the program randfold determined that lncRNAs with TEs form more stable secondary structures than those without TEs [127]. Another line of supporting evidence came from the analysis of A to I editing sites in lncRNAs, which modulates the base pairing of the dsRNA. It was found that about 82% of RNA editing sites locate in the *Alu* regions of lncRNAs. This suggests the *Alu* regions in regulatory RNAs are involved in inter- and intramolecular base pairing to form stable secondary structures [127].

In summary, the findings of different studies indicate a clear role of TEs in the functionality of regulatory RNAs in different ways, including, but not limited to, helping the circularization of circular lncRNAs, binding of regulatory RNA to target mRNAs, and formation of the stable secondary structure of regulatory RNAs.

### 3.3. Role of TEs in Lineage Specificity of Regulatory RNAs

Several studies have reported the lineage-specificity of TE-derived regulatory RNAs. For example, the work by Piriyapongsa et al. [122], which examined the per-site conservation scores of miRNA sequences in the miRbase data, showed that, on average, TE-derived miRNAs are less conserved than non-TE-derived miRNAs. Out of 55 TE-derived miRNAs, only 18 were found as conserved (conservation score above a fixed threshold), and 37 were non-conserved. The least-conserved ones were primate-specific [122]. As another example, a placental-specific miRNA gene family mir-1302 has all its members derived from MER53 transposons (eutherian-specific TE) with 58 potential orthologs in placental mammals, indicating the emergence of this miRNA family after the placental mammals diverged from marsupials [119]. As shown in another study by Qin et al. (2015), the proportions of TE-derived miRNA increased with the evolution of vertebrates from less than 5% in zebrafish to ~20% in humans. Further, sequence analysis of these miRNAs shown no homology among these TE-derived miRNAs from *Danio rerio*, *Gallus gallus*, and mammals, indicating that TE-derived miRNAs were lineage-specific due to lineage-specific TE transpositions [124].

lncRNAs have a significant role in the evolution of key regulatory networks underlying the evolutionary processes [143]. TEs likely have contributed to the functional evolution of lncRNA genes [142]. The insertion of TEs in lncRNA genes is considered as an important mechanism behind lineage-specific changes in lncRNAs-mediated gene regulation. Primate-specific TEs were identified in the known TSSs of eight functionally characterized lncRNAs, suggesting the role of TEs in the birth of these lncRNAs during primate evolution [127]. Another study by Kannan et al. determined the evolutionary rate of human lncRNAs by estimating pairwise evolutionary distances for human–macaque alignment and found a significant positive correlation between TE content and the evolutionary rate of lncRNAs [137]. As an example, in the case of *Xist* lncRNA, many TEs are already present in the *Xist* locus of the Eutherian ancestor involved in the generation of the first functional *Xist* transcript. However, many other TEs in the *Xist* exons are lineage-specific and contribute to *Xist*’s functional diversification during Eutherian evolution [18].

In summary, TE-derived regulatory RNAs tend to be less conserved and lineage-specific, implicating TEs as an important source of lineage-specificity of regulatory RNAs.

### 3.4. Tissue-Specificity of TE-Derived Regulatory RNAs

Beyond lineage-specificity, studies have also shown that TE-enriched regulatory RNAs can be tissue-specific. For example, in the study by Kang et al., a total of 29 human lncRNAs were found to have tissue-specific expression, out of which 20 were TE-derived lncRNAs. Moreover, 9 of the 11 lncRNAs found to be expressed in cancer cell lines contain TE sequences, indicating the role of TE-embedded lncRNAs in cancer [128]. In another study, it was observed that 127 human lincRNAs containing HERV-H sequences were expressed at much higher levels in pluripotent cells, H1-hESCs, and iPSCs, with HERVH LTR in the TSSs of the lncRNA genes, suggesting that TEs might induce tissue-specific expression in these cases [136]. The TE-driven tissue-specific expression of lncRNAs has been further elucidated in the study by Chishima et al. (2018), which identified many TE–tissue pairs associated with tissue-specific expression of lncRNAs using tissue expression data of human lncRNAs from three different datasets of ‘Expression Atlas’. For example, ERV1-lncRNAs were shown to express specifically in testis and L1PA2 was shown to promote the placental specific expression of L1PA2-lncRNAs with the antisense promoter of L1PA2 overlapping with the TSS-neighboring region of lncRNAs, being the likely driver of tissue-specific expression [144].

In summary, regulatory RNAs with embedded TE sequences have been revealed to have tissue-specific expression patterns, and, in some cases, TEs in the TSS neighboring region of lncRNAs may be responsible for driving tissue-specific expression.

### 3.5. Differential Contribution to Regulatory RNAs among TE Types

Different types of TEs have a varying contribution to human regulatory RNA sequences. For miRNAs, the study by Qin et al. (2015) classified TE-derived human miRNAs from miRbase in three different types and found (1) SINEs and LINEs are the major contributors to miRNA sequences with inverted TE sequences; (2) SINEs, LINEs, and DNA transposons are major contributors to miRNAs with partial overlaps with non-inverted TE sequences; (3) DNA transposons and SINEs are the primary contributors to miRNA derived entirely from TEs. LTR retrotransposons were thus found to have the least contribution in all three types of miRNAs [124].

Several studies also examined the TE composition of human lncRNAs. A study found that SINEs and LINEs as the prevalent TE types contribute 29% of the sequences for the 7700 TE-derived lincRNAs, despite shown as depleted compared to their genome averages (L1s depleted by 2-fold and *Alu* elements depleted by 1.4-fold), while LTR families were showed to be enriched in these lncRNAs despite not being a major TE contributor [136]. Kang and coworkers found that 61 of the 94 human lncRNA sequences from lncRNAdb had TEs, most belonging to SINEs and LINEs. The percentage of lncRNA sequence contributed by different types of TEs was 13% for LINEs, 7.7% for SINEs, 3.5% for LTRs, and 2.2% for DNAs, with *Alu*Sx and L1 subfamilies having the highest copy number [128]. Thus, both of the above studies showed that SINEs and LINEs contribute most to the lncRNA sequences, but in less proportion compared to their contribution in the whole-genome. This is further supported in the study by Kapusta and coworkers, which in the analysis of human lncRNA sequences from Gencode, showed that LINEs were under-represented and LTRs were over-represented in lncRNA sequences (~30% vs. ~40% for LINEs and 30% vs. 20% for LTRs in the lncRNAs vs. the genome, respectively). Further, LTRs were over-represented in the exonic and proximal region of lncRNA genes than that of protein-coding genes [127]. In another study, different regions of lincRNA genes from—non-encoding RNA expression database (NRED) in the human genome were analyzed to assess the contribution of different TE types. It was observed that the distribution of TEs in the introns of lincRNA genes was similar to that in the whole-genome, indicating no bias for specific TE type. However, there was a significant reduction of LINEs in exonic and promoter regions of lincRNA genes (~5% vs. ~20% in the whole-genome), likely due to their deleterious impact when inserted into the functional regions of genes [137].

From the findings of the studies mentioned above, it can be said concludingly that among all TEs, SINEs and LINEs contribute most to the lncRNA sequence. However, in contrast to the whole-genome, SINEs and LINEs are under-represented, while LTRs are overrepresented in lncRNAs. In summary, TEs’ distribution in introns of lncRNA genes is roughly similar to that of the whole-genome, but in exonic and promoter regions, LINEs are under-represented, while LTRs are over-represented in the exons and promoters of lncRNAs in comparison with protein-coding genes.

## 4. Summary and Perspectives

**Figure 2:**
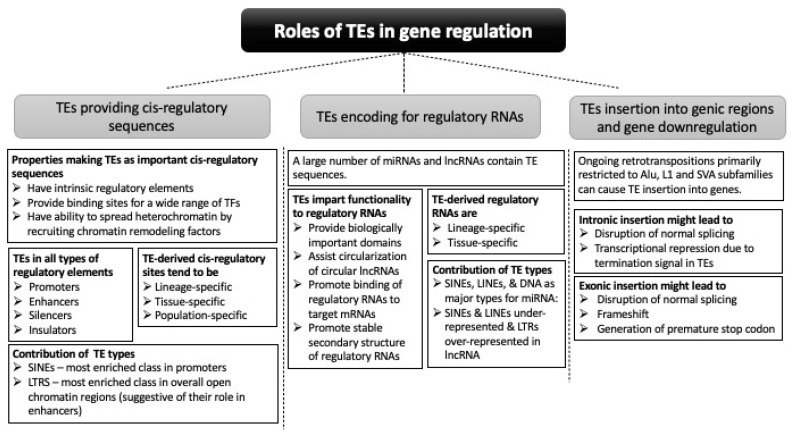
Different ways by which TEs contribute to gene regulation.

This review considers two aspects of TEs’ contribution to gene regulation: in cis-regulatory sequences and in regulatory RNAs (Figure 2).

TEs have intrinsic regulatory properties for regulating their own expression and provide ready-to-use TFBSs or undergo mutations to provide binding motifs for TFs. TE sequences have been found in the regulatory elements of many genes, participating in short-range and long-range control of gene expression. Among different classes of TEs, SINEs have the highest contribution in all types of regulatory regions. Genes with tissue-specific expression are more likely to have TE sequences in the regulatory regions. TE-derived regulatory sites tend to be lineage-specific as well as species-specific. Furthermore, polymorphic TEs have been associated with gene expression differences among populations or even individuals.

TEs also contribute to gene regulation by directly participating in the generation of regulatory RNAs. Some TE types are associated explicitly with certain miRNA families. TE sequences in the regulatory RNAs are crucial for their regulatory function by assisting in the formation of secondary structures of regulatory RNAs and in the binding of regulatory RNAs to their target mRNA sequences. TEs also provide sequence and structural motifs to regulatory RNAs that facilitates the interaction with other biological molecules. Like the TE-derived cis-regulatory sequences, TE-derived regulatory RNA sequences tend to be lineage-specific as well. Furthermore, the tissue-specific expression of TE-derived regulatory RNAs has started to be recognized. Among different types of TEs, SINEs and LINEs contribute most to lncRNA sequence, and DNA transposons and SINEs are the major contributors for miRNAs entirely derived from TEs.

Research on TEs’ role in gene regulation is still in its early-stage, leaving ample room for further investigation. For example, systematic studies are needed to comprehensively unveil the contribution of different TE types in the cis-regulatory regions and regulatory RNA sequences using databases providing the most recent annotations. Moreover, there is a need to comprehensively analyze the evolutionary dynamics of these TE-derived regulatory elements genome-wide, instead of just focusing on particular subsets. Additionally, there is a need to correlate polymorphisms of TE-derived regulatory elements with the different gene expression patterns among populations and even individuals. Such types of studies demand specialized datasets providing genotype calls of the TEs present in regulatory regions and matching gene expression data of the same individuals in more diverse tissues. Experimental verification of the functional impact of TEs on gene regulation is also essential.

## Figures and Tables

**Table 1:** Comprehensive list of major primary and secondary gene regulation databases.

Primary Databases			
Database	Brief Description	Specie	Reference
Encyclopedia of DNA Elements (ENCODE)	Provides following functional genomics data for the diverse range of tissues and cell lines: DNase-seq data, FAIRE-seq data, Histone ChIP-seq data, TF ChIP-seq data	Human	[36]
Roadmap Epigenomics Mapping Consortium (REMC)	Provides following functional genomics data for the diverse range of tissues and cell lines: DNase-seq data. Histone ChIP-seq data, WGBS data, RRBS data	Human	[30]
Genomics of Gene Regulation (GGR)	The database is limited to only A549 cell lines and few primary cells. Provides following functional genomics data: DNase-seq data, Histone ChIP-seq data, TF ChIP-seq data	Human, mouse	[39]
Blueprint epigenome project	Provides reference epigenomes of distinct types of hematopoietic cells. Includes following functional genomics data: DNase-seq data, Histone ChIP-seq data, WGBS data	Human	[37]
**Secondary Databases**			
**Database**	**Brief Description**	**Specie**	**Reference**
Open Chromatin Database (OCHROdb)	Integrates DNase seq data from ENCODE, Roadmap Epigenomics, Genomics of Gene Regulation and Blueprint Epigenome to provide a comparison of open chromatin regions across multiple samples	Human	[40]
ChIPSummitDB	Determines cistrome of TFs by analyzing TF ChIP-seq data from primary databases	Human	[41]
Super-enhancer database (SEdb)	Maps super-enhancer regions in the genome by analyzing ChIP-seq data of H3K27ac. The current version documents a total of 331,601 super-enhancers from 542 samples	Human	[38]
EnhancerAtlas	Identifies enhancer region by integrating datasets of 12 high-throughput methods. In contrast to other enhancer databases (SEdb, HACER, REdb, HEDD, DiseaseEnhancer, TiED, GeneHancer, SEA, DENdb and dbSUPER), it combines a versatile and most comprehensive set of annotations	9 species, including human	[29]
Genome Segmentations from ENCODE data	Identifies functional regulatory elements in the genome by integrating ChIP-seq data for 8 chromatin marks, RNA polymerase II, the CTCF transcription factor. It involves the application of two unsupervised machine learning techniques (ChromHMM and Segway) to assign genomic states to disjoint segments in the genome	Human	[42,43]
Cistrome Data Browser (Cistrome DB)	Combines raw ChIP-seq and chromatin accessibility data from ENCODE, Roadmap and few other resources and process it through the same pipeline and quality control metrics to achieve consistency and provides a dataset with standardized curation, quality control and analysis procedures	Human, mouse	[44]
